# Determinants of Exposures to Hazardous Materials among Nail Cosmeticians in the Kampala City, Uganda

**DOI:** 10.1155/2019/1925863

**Published:** 2019-04-01

**Authors:** John C. Ssempebwa, Rawlance Ndejjo, Ruth Mubeezi Neebye, Edwinah Atusingwize, Geofrey Musinguzi

**Affiliations:** Department of Disease Control and Environmental Health, School of Public Health, College of Health Sciences, Makerere University, P.O. Box 7072, Kampala, Uganda

## Abstract

Globally, nail salons represent a fast expanding industry and often with low-income cosmeticians. In general, cosmeticians have limited access to safety information about the hazardous materials they handle, which would potentially enable them to minimize workplace exposures. The problem is much pronounced in low- and middle-income countries due to weaknesses in regulation of the industry. We investigated determinants of exposures to hazardous materials among nail cosmeticians in Kampala District, Uganda. We employed a cross-sectional study design among a random sample of 243 participants. The sociodemographic characteristics, education and training status, knowledge about routes of exposure to hazardous chemicals, and personal protective material use of cosmeticians were assessed through face-to-face interviews. Most cosmeticians were aged 18–34 years, and more males were engaged in this work than females. Also, 82.7% believed inhalation was the major exposure route for the chemicals they handled. Participants who had attained secondary-level education and above were over three times more likely to wear masks (AOR = 3.19, 95% CI 1.58–6.41) and gloves (AOR = 3.48, 95% CI 1.55–7.81) and over two times more likely to use aprons (AOR = 2.50, 95% CI 1.18–5.32). Participants who had ever received safety training on hazardous chemicals were more likely to wear all four personal protective equipment: masks (AOR = 3.21, 95% CI 1.61–6.42), gloves (AOR = 4.23, 95% CI 2.05–8.75), goggles (AOR = 4.14, 95% CI 1.25–13.65), and aprons (AOR = 2.73, 95% CI 1.25–5.96). Participants who had spent more than two years in the nail cosmetics business were more likely to wear masks (AOR = 3.37, 95% CI 1.64–6.95). With the increasing demand for nail cosmetics, and many people in urban areas of low-income countries engaging in this industry, there is need for training and better workplace policies to promote a healthier urban workforce dealing in cosmetics.

## 1. Introduction

Beauty salons are workplaces where a number of activities including beauty treatments for skin, hair, and nails take place. Most of these activities particularly for nails involve the use of chemicals whose nature is often hazardous [1–3]. Nail cosmetics include the application of nail polish, artificial nails, nail elongations, and other nail treatments as fashion trends may influence. The popularity of nail cosmetics is on the rise globally and especially in urban areas [2, 4–7], and with the increasing number of clients, beauty salon workers have a risk of increased and prolonged exposures to various hazardous materials in the workplace [8–10]. Many products are used during manicures and pedicures, and depending on the treatment being performed, exposure and health risks will vary amongst nail cosmeticians. Among them, the products used are enamel, adhesives, polishes, and polish removers [11, 12]. Nail enamel and polish removers are strong solvents, which are highly volatile [13, 14]. Exposure to such organic solvents can cause many problems including irritation to nose, throat, lung, skin, and eyes [15–17]; headaches; lightheadedness; nausea; and confusion [18]. Nail polish may contain formaldehyde and some methacrylates which can cause occupational asthma with repeated inhalation exposure [19–22]. Such substances may also aggravate preexisting asthma [20, 23]. Ethyl methacrylate and other related methacrylates that replaced methyl methacrylate used to attach the artificial piece to the nail were banned because of their potential for sensitizing and are documented to cause occupational asthma and contact dermatitis [24] and fungal infections [21] in some workers. Indeed, evidence shows that several environmental exposures to chemical mixtures may adversely affect health [25].

In many low-income countries, salons use cheap cosmetics to keep the costs as low as possible [26–28], without much concern for health risks. Nail cosmeticians are consequently at increased risk because they handle the chemicals on a daily basis; therefore, it is important that they become aware of the hazards. In Kampala, Uganda, the nail cosmeticians do not restrict their activities to beauty salons; some are mobile, often moving with an assortment of chemicals and wares for their trade. These mobile nail bars are set up to serve customers interested in nail beautification conducted at a place of their convenience.

In this study, we focused on salons and mobile nail bars that offered manicure and pedicure services in Kampala, Uganda. The study assessed knowledge and practices of nail cosmeticians, and other factors associated with exposures to hazardous materials at their workplaces in the Kampala city, to provide baseline information aimed at improving their safety and health. The study probed for the nature of chemicals used, their handling, presence, and use of protective wear. Self-reported direct health effects such as headaches and skin irritations such as itchiness, swelling, burning sensation, and allergic reaction were also investigated.

## 2. Methods

### 2.1. Study Area and Population

**Figure 1:**
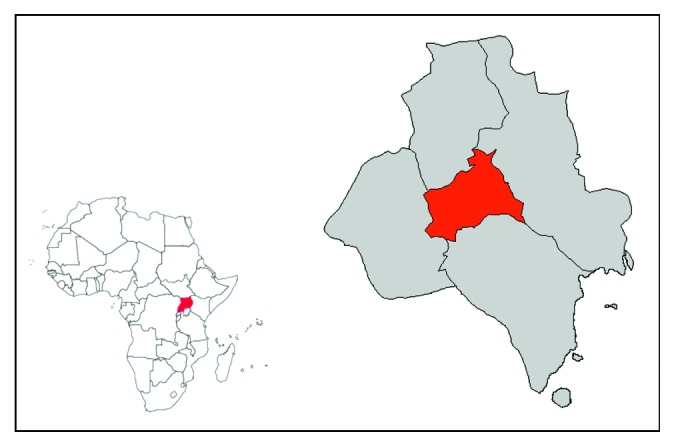
Map of Africa showing Uganda and Kampala district administrative divisions (source: Uganda Bureau of Statistics, 2007).

The study was conducted in the parishes within the five administrative divisions of the Kampala city, namely, Kampala Central, Kawempe, Makindye, Nakawa, and Rubaga divisions, as indicated in Figure 1. The study population was comprised nail cosmeticians in beauty salons and mobile nail bars. Nail cosmeticians working in the Kampala city and having worked for more than three months were eligible.

Approval to conduct this study was sought from the Higher Degrees, Research and Ethics Committee of Makerere University School of Public Health and the Uganda National Council for Science and Technology. Informed consent was obtained from the respondents before their participation in the study.

### 2.2. Study Design

A cross-sectional design using quantitative methods of data collection was employed. Trained research assistants used pretested structured questionnaires together with observational checklists to obtain the data. The face-to-face interviews took place at the workplace to enable the participants keep working where possible during the interview sessions.

### 2.3. Sample Size and Sampling Procedure

Using the Leslie Kish formula (1965), a sample size of 243 nail cosmeticians was selected from the five divisions for the quantitative data. Multistage sampling was performed, whereby names of zones in each division were written on pieces of paper and put in different boxes. Using simple random sampling with replacement, two zones in each division were selected from the boxes. The number of salons in the zones ranged from 40 to 100. In each zone, a central point was established from which the first salon was randomly selected and then the subsequent ones systematically selected using a set interval. Where there were more than one eligible participant within a selected salon, one was randomly selected to answer the study questionnaire while neighbouring salons were selected to replace those without any eligible participants. Observational checklists were used to collect data on the participants' practices in relation to occupational safety and health.

### 2.4. Data Analysis

The study had two outcome variables, namely, knowledge of respondent about routes of exposure to cosmetics-related hazardous chemicals and use of personal protective wear or equipment (PPE) among nail cosmeticians. To obtain the outcome variable for knowledge, knowledge scores were generated by assigning a score of 1 to each of the 11 knowledge questions correctly answered by the respondent. Participants with scores above 70% were categorized as knowledgeable and assigned 1 and the rest assigned 0 forming a binary outcome. For personal protective equipment, the use of each of the four wears, namely, mask/handkerchief, gloves, goggles, and aprons, were independently assessed. Participants who reported using each of the equipment/wear were assigned 1 and those who reported not using it were assigned 0 to form a binary outcome variable for each of the personal protective equipment. The outcome variables were then run against the exploratory variables and associations established. For each outcome variable, all variables from the bivariate analysis were put into the same model at multivariate analysis and a binary logistic regression using the backward elimination method performed to ascertain the independent predictors of a given relationship. Odds ratios were used as measures of association, and a *P* value of <0.05 was considered for a statistically significant relationship at the 95% confidence interval. Quantitative data were entered using EpiData version 3.02 (EpiData Association, Denmark) and analyzed in Stata 13.0 (StataCorp, Texas, USA) software.

## 3. Results and Discussion

### 3.1. Sociodemographic Characteristics of Respondents

Majority, 225 (92.6%), were aged between 18 to 34 years, males 194 (79.8%) and not married 157 (64.6%) Table 1. In a study done in Burkina Faso, the mean age of pedicure-manicure practitioners was 19 years with the age range of 25–35 years [29]. It is probable that after attaining the mandatory working age of 18 years, many perceive pedicure-manicure as a simple to engage in work sector. In a study done in Boston, USA, the mean age of nail cosmeticians was 34, with an age range 17–55 years [16] slightly lower than that in Samaru, North Western Nigeria, where the mean age was 40 years, with the age range 27–65 [30]. These findings suggest contextual differences, which could be probably explained by the changing economies where young people end up in small entrepreneur businesses [31]. Globally, women dominate this industry [29, 32–34]; however, in the study, this was the reverse; more men are resorting to less manual work or due to lessening job opportunities, and men are seeking work in this employment sector. Over half, 143 (58.9%), had attained secondary education and above and had worked in the nail cosmetics business for over two years (153 (63%)). Most (60%) had never got any training on the handling of hazardous chemicals they used for their work. This situation is common in many other countries where the nail cosmeticians do not have any training concerning the materials they handle and apply consequently posing a risk to both themselves and their clients [29, 35].

Almost all nail cosmeticians (91%) did not belong to any association that brings together operators engaged in this trade. This is a challenge for the trade in that such associations would work to bring together the cosmeticians as a central arm to ease access to health education and information. In many low-income countries where workers associations exist, cosmeticians do not join them or are not aware of them [36], missing benefits such groups bring as seen in some high-income countries [37].

### 3.2. Nail Cosmetician's Knowledge about Routes of Exposure to Cosmetics-Related Hazardous Chemicals

To assess the level of knowledge of nail cosmeticians about the routes of exposure of chemicals found in nail cosmetics, they were subjected to a set of 11 questions and were required to respond with a “Yes”, “No,” or “Don't know.” The majority indicated that chemicals entered the body by inhalation (201 (82.7%)) and ingestion (183 (75.3%)). Additionally, most (209 (87.1%)) indicated that use of gloves provides a protective barrier against entry of chemicals into the body (Table 2). The other possible routes of exposure were not as much recognized.

This is of public health significance especially because chemicals can be absorbed through the skin [38–41]. In this study, unlike in Benin City where 69.0% of beauticians did not know that their occupation had an effect on their health [9], the participants knew and only differed on the routes of exposure.

### 3.3. Knowledge of Nail Cosmeticians about Routes of Exposure to Cosmetics-Related Hazardous Chemicals

Using an ordinal score, the itemized responses of the knowledge questions were ranked to categorize participants as either knowledgeable or less knowledgeable. The results showed that 62 (25.5%) were knowledgeable. Most of those knowledgeable were males (48 (77.4%)) and had attained secondary education and above (38 (61.3%)). The crude and adjusted odds ratios revealed that sociodemographic variables did not predict the Nail cosmeticians' knowledge about exposure to cosmetics-related hazardous chemicals. Although not seen in this study, nail cosmeticians greatly improve their knowledge and benefit from being trained on the safe handling of the chemicals they use [2, 5, 35, 42–44]. The training would also need to be delivered in the workers local language [2, 35].

### 3.4. Cosmetics and Personal Protective Equipment Commonly Used by Nail Cosmeticians

The most commonly used cosmetics were nail polish (238 (97.9%)), polish removers (239 (98.4%)), and artificial nails (201 (82.7%)). For personal protective equipment, most used aprons (175 (72.3%)) and masks that covered the nose and mouth (120 (49.4%)) (Table 3).

About half the participants reported using masks or improvised with handkerchiefs to cover the nose and mouth while working. It was noted that the type of masks they used were dust masks and were not appropriate for protection from chemical vapours. In addition to the inappropriate wear, the use was also infrequent as they cited unavailability (46%) and being unnecessary (33%) or uncomfortable (35%). The reported nonuse of about 50% is quite high compared to other studies in the USA ranging between 10 and 32% [16, 45, 46]. Our findings show common use of inappropriate protective wear as previously reported in Boston, where surgical masks were used by nail salon workers as personal protective equipment which do not protect the user from chemical vapours and dust [47, 48].

Similar to our study, other studies have reported nonuse of gloves among nail cosmeticians at 30–74% in the USA [16, 46, 49] and 95% in Brazil [50]. The figures show a relatively more adherence to use of masks than of gloves globally. This could be a perception that there is less risk of dermal exposure than of inhalation. It could also be due to the unease of working while wearing gloves. Absence of skin disorders has been associated with glove use [16]. In our study, inaccessibility to gloves at the salon was a major reason for nonuse.

Use of goggles for eye protection was very rare (7%). Appropriate goggles are not readily available in the study area, and those available are quite costly ($0.25–1 a pair), which is relatively expensive compared to the service fees charged in the salons. Aprons were the most used PPEs (72.3%) by the cosmeticians, which is similar to findings in other studies in USA [51] and Iran [52]. Many aprons were of washable fabrics such as cotton and polyester, and others were plastic disposable ones although these were being reused.

### 3.5. Factors Associated with Use of Personal Protective Equipment among Nail Cosmeticians

At bivariate analysis (Table 4), the participants' level of education was significantly associated with the use of masks (COR = 2.92, 95% CI 1.72–4.99), gloves (COR = 3.09, 95% CI 1.68–5.69), and aprons (COR = 3.48, 95% CI 1.93–6.26). Participants who had ever received training on safe handling of hazardous chemicals were more likely to wear PPEs: masks (COR = 3.23, 95% CI 1.86–5.61), gloves (COR = 4.49, 95% CI 2.51–8.05), goggles (COR = 3.43, 95% CI 1.22–9.63), and aprons (COR = 2.92, 95% CI 1.50–5.66). In addition, the registration status of the salon was significantly associated with the use of masks (COR = 2.69, 95% CI 1.60–4.53); gloves (COR = 2.61, 95% CI 1.49–4.58); and aprons (COR = 5.05, 95% CI 2.61–9.77). Female participants were also more likely to use gloves (COR = 4.11, 95% CI 2.14–7.91) and aprons (COR = 4.16, 95% CI 1.57–11.02). Participants who had spent more than two years in the nail cosmetics business were more likely to use masks (COR = 2.65, 95% CI 1.54–4.54).

When all significant variables from the bivariate analysis were transferred to a multivariable logistic regression model and the effect of potential confounders controlled for (Table 4), the level of education, training on safety measures, gender, duration, and registration maintained significance although some had changes in the observed effect. Participants who had attained secondary-level education and above were over 3 times more likely to wear masks (AOR = 3.19, 95% CI 1.58–6.41) and gloves (AOR = 3.48, 95% CI 1.55–7.81) and over two times more likely to use aprons (AOR = 2.50, 95% CI 1.18–5.32). Participants who had ever received safety training on hazardous chemicals were more likely to wear all four personal protective equipment: masks (AOR = 3.21, 95% CI 1.61–6.42), gloves (AOR = 4.23, 95% CI 2.05–8.75), goggles (AOR = 4.14, 95% CI 1.25–13.65), and aprons (AOR = 2.73, 95% CI 1.25–5.96).

Education has been reported to enhance PPE use among workers in nail salons [53, 54]. Education demystifies beliefs and perceptions. As documented in other studies, most nail cosmeticians learned how to do nails on the job [55, 56]. As a result, the practice of how to protect oneself initially depends on the practice of who is doing the teaching at the salon. However, more often, workers are not taught about the risks of exposures or any steps that they could take to protect themselves.

The training received on handling of hazardous chemicals shows to have improved on the use of all the four PPEs investigated as shown elsewhere [2]. This signifies the necessity of education for workers to appreciate use of PPE in a work environment. Like in hair salons [57], the foundation for managing nail cosmetic problems is prevention through education. Familiarity with the procedures and materials used in the industry is essential for safe nail care strategies, which calls for regular training of workers engaged in the nail industry. However, use or reluctance and/or failure to use PPEs is not only influenced by the training but also their cost and availability on the market and employment conditions. It has also been reported that masks were not likely to be used because of their appearance [47].

Although their statistical significance was lost at the multivariate level, the probability of wearing aprons and gloves remained high among women than men. Females were only significantly less likely to wear masks than males (AOR = 0.29, 95% CI 0.12–0.70). Females take more care of their hands and clothes than males, and probably that is why they make more effort to wear gloves where they are available and also to keep their clothes clean. Further, while the significance of registration status of the salon was lost for gloves, it remained for wearing of masks (AOR = 3.95, 95% CI 1.68–9.30) and aprons (AOR = 2.59. 95% CI 1.03–6.55). The Public Health Act of Uganda (2000) requires all business owners to acquire certificates of suitability before being granted trading licenses. It is therefore a legal requirement prior to premise registration that PPEs are available for inspection. However, once registration is done, there is often a tendency to relax, for instance, on replacing PPEs as necessary. Moreover, the Occupational Safety and Health Act (2006) requires employers to supply their employees with PPE for their use. However, many salon owners also perceive the costs of PPEs as a barrier to their use and not as a necessity in protecting health. Indeed, assessments conducted by the city authority showed that while many salon employees had no certificates of suitability, they were operating. This implies there are nail cosmeticians that may not be suitable to engage in the nail business. This suggests that the safety and health standard of salons in Kampala is poor.

Participants who had spent more than two years in the nail cosmetics business were more likely to wear masks (AOR = 3.37, 95% CI 1.64–6.95). The length of time spent in a given employment teaches a lot to a worker. Over the years, workers are able to learn through their experiences and may therefore be better positioned to adapt to PPE use.

### 3.6. Limitations

The study solely focused on knowledge of the chemical components, and yet, there are complications that may occur during or subsequent to the nail-grooming procedure. These include various physical exposures including ergonomics, dust or respirable particulate matter, and pollutants of outdoor origin. Also, responses were self-reported except for the observation questions, and this could be associated with bias. Nevertheless, the study provides essential information that can be used as a basis for strategies to improve protection of nail technicians in the Kampala city and similar settings.

## 4. Conclusions and Recommendations

In this study, multiple chemicals were handled in the nail cosmetics used, and most cosmeticians believed inhalation was the major exposure route for the chemicals they handled. Some cosmeticians were underinformed about the chemicals they handle, due to instructions being in a foreign language, which made it hard to understand. They also lacked training on handling hazardous chemicals. Gender, level of education, the training on safety, registration status of salon, and duration in business were significantly associated with use of different PPEs among the nail cosmeticians. Since there is increasing demand for nail cosmetics, and many people in low-income countries such as Uganda are engaging in this industry, there is need to advocate for all nail cosmeticians to attend vocational training including hazardous material handling and protective measures.

## Figures and Tables

**Table 1:** Sociodemographic characteristics of participants.

Characteristics of participants (*n*=243)	Frequency (%)
*Age (years)*	
18–34	225 (92.6)
35–60	18 (7.4)

*Sex*	
Male	194 (79.8)
Female	49 (20.2)

*Marital status*	
Single	157 (64.6)
Married/cohabiting	86 (35.4)

*Level of education*	
Primary	100 (41.1)
Secondary and above	143 (58.9)

*Religion*	
Christians	202 (83.1)
Muslim	41 (16.9)

*Duration in nail cosmetics business*	
Two years or less	90 (37.0)
More than two years	153 (63.0)

*Salon registered*	
Yes	114 (46.9)
No	129 (53.1)

*Ever received training on safety of handling hazardous chemicals*	
Yes	88 (37.0)
No	150 (63.0)

*Average number of nail cosmetics types used*	
Five or less	136 (56.0)
Over five	107 (44.0)

*Nail technician belongs to city beauty operators association*	
Yes	21 (9.0)
No	221 (90.9)

**Table 2:** Knowledge of nail cosmeticians about exposure routes to cosmetics-related hazardous chemicals.

Knowledge prompt: chemicals from nail cosmetics (*n*=243)	Yes (%)	No (%)	Don't know (%)
Can enter the body through breathing them in	201 (82.7)	27 (11.1)	15 (6.2)
Can enter the body through ingesting them	183 (75.3)	42 (17.3)	18 (7.4)
Can enter the body via contact with contaminated surfaces	97 (40.1)	102 (42.1)	43 (17.8)
Can enter body through contact with spills and splashes	90 (37.8)	84 (35.3)	64 (26.9)
In gas or vapour form can enter body through skin	129 (53.7)	77 (32.1)	34 (14.2)
In dry form have potential to be absorbed through skin	99 (40.9)	88 (36.4)	55 (22.7)
In liquid form can be absorbed through skin	146 (61.1)	64 (26.8)	29 (12.1)
Can penetrate any part of the body skin	96 (39.7)	94 (38.8)	52 (21.5)
Can be protected against by using gloves	209 (87.1)	26 (10.8)	5 (2.1)
Can move from skin into the body during washing of hands	74 (30.6)	152 (62.8)	16 (6.6)
Can taint food if hands are not washed	195 (81.2)	42 (17.5)	3 (1.2)

Multiple responses were given.

**Table 3:** Commonly used nail cosmetics and personal protective equipment use among nail cosmeticians.

Practice	Frequency, (*n*=243) (%)
*Cosmetics used by nail cosmeticians*	
Nail polish	238 (97.9)
Polish remover	239 (98.4)
Nail elongators	65 (26.7)
Artificial nails	201 (82.7)
Nail enamel	95 (39.1)
Nail filing	185 (76.1)
Adhesives	162 (66.7)
Personal protective equipment used by nail cosmeticians while working	

*Mask/handkerchief*	
Yes	120 (49.4)
No	123 (50.6)

*Gloves (hand and arm protection)*	
Yes	75 (31.1)
No	166 (68.9)

*Goggles (eyes protection)*	
Yes	17 (7.0)
No	226 (93.0)

*Apron (general protection)*	
Yes	175 (72.3)
No	67 (27.7)

*Reason for infrequent use of PPE*	
Unavailable	111 (45.7)
Unnecessary	80 (32.9)
Uncomfortable	86 (35.4)
Nobody else uses them	13 (5.3)
Unaware	11 (4.5)

*Cosmetics used have instructions for use*	
Yes (always)	174 (71.6)
Sometimes	29 (11.9)
No	35 (14.4)
Didn't know	5 (2.1)

*Able to read and understand the instructions and content label (n*=203)	
Yes	135 (66.5)
No	37 (18.2)
Sometimes	31 (15.3)

*Why find it hard to understand instructions or content label* (*n*=54)	
Instructions in foreign language	45 (83.3)
Instructions in small prints	5 (9.3)
The label is full of chemical formula and names	4 (7.4)

Multiple responses were given.

**Table 4:** Crude associations for factors associated with use of personal protective equipment among nail cosmeticians.

Characteristics	Used mask		Used gloves		Used goggles		Used aprons	
	COR (95% CI)	AOR (95% CI)	COR (95% CI)	AOR (95% CI)	COR (95% CI)	AOR (95% CI)	COR (95% CI)	AOR (95% CI)
*Age (years)*								
18–34	1	—	1	—	1	—	1	—
35–60	2.17 (0.78–5.97)	2.98 (0.69–12.91)	4.58 (1.63–12.92)^b^	2.18 (0.65–7.34)	3.01 (0.78–11.66)	3.07 (0.51–18.29)	7.10 (0.93–54.4)	5.17 (0.55–48.75)

*Gender*								
Male	1	—	1	—	1	—	1	—
Female	0.52 (0.27–0.99)	0.29 (0.12–0.70)^c^	4.11 (2.14–7.91)^a^	2.29 (0.98–5.35)	1.24 (0.38–3.98)	0.87 (0.22–3.39)	4.16 (1.57–11.02)^b^	2.59 (0.83–8.02)

*Marital status*								
Single	1	—	1	—	1	—	1	—
Married/cohabiting	1.20 (0.71–2.03)	1.14 (0.54–2.38)	1.27 (0.72–2.24)	—	0.99 (0.35–2.79)	0.55 (0.14–2.11)	1.05 (0.58–1.89)	1.41 (0.65–3.07)

*Level of education*								
Primary	1	—	1	—	1	—	1	—
Secondary and above	2.92 (1.72–4.99)^a^	3.19 (1.58–6.41)^b^	3.09 (1.68–5.69)^a^	3.48 (1.55–7.81)^b^	0.99 (0.37–2.72)	0.73 (0.22–2.48)	3.48 (1.93–6.26)^a^	2.50 (1.18–5.32)^c^

*Religion*								
Christians	1	—	1	—	1	—	1	—
Muslim	1.09 (0.56–2.14)	2.01 (0.87 (4.66)	0.89 (0.43–1.88)	—	0.29 (0.04–2.25)	0.36 (0.04–3.02)	1.44 (0.65–3.21)	2.19 (0.84–5.70)

*Duration in nail cosmetics business*								
Two years or less	1	—	1	—	1	—	1	—
More than two years	2.65 (1.54–4.54)^a^	3.37 (1.64–6.95)^b^	1.09 (0.62–1.91)	1.17 (0.55–2.48)	1.08 (0.39–3.04)	0.95 (0.28–3.15)	0.58 (0.31–1.07)	—

*Salon registered*								
No	1	—	1	—	1	—	1	—
Yes	2.69 (1.60–4.53)^a^	3.95 (1.68–9.30)^b^	2.61 (1.49–4.58)^b^	1.85 (0.83–4.14)	1.67 (0.62–4.56)	0.81 (0.22–2.97)	5.05 (2.61–9.77)^a^	2.59 (1.03–6.55)^c^

*Ever received training on safe handling of hazardous chemicals*								
No	1		1		1		1	
Yes	3.23 (1.86–5.61)^a^	3.21 (1.61–6.42)^b^	4.49 (2.51–8.05)^a^	4.23 (2.05–8.75)^a^	3.43 (1.22–9.63)^c^	4.14 (1.25–13.65)^a^	2.92 (1.50–5.66)^b^	2.73 (1.25–5.96)^c^

*On average, how many types of nail cosmetics do you use*								
Five or less	1	—	1	—	1	—	1	—
Over five	2.28 (1.36–3.82)^b^	1.51 (0.72–3.19)	2.14 (1.23–3.73)^c^	1.19 (0.52–2.76)	1.14 (0.795–3.06)	1.09 (0.29–4.09)	1.22 (0.69–2.16)	1.43 (0.63–3.24)

*Salon registered*								
No	1	—	1	—	1	—	1	—
Yes	2.69 (1.60–4.53)^a^	3.95 (1.68–9.30)^b^	2.61 (1.49–4.58)^b^	1.85 (0.83–4.14)	1.67 (0.62–4.56)	0.81 (0.22–2.97)	5.05 (2.61–9.77)^a^	2.59 (1.03–6.55)^c^

*Do you belong to city beauty operators association*								
No	1	—	1	—	1	—	1	—
Yes	3.00 (1.13–7.95)^c^	—	1.29 (0.52–3.24)	0.70 (0.22–2.23)	1.37 (0.29–6.44)	—	1.80 (0.59–5.55)	1.95 (0.53–7.19)

^a^ *P* < 0.001; ^b^*P* < 0.005; ^c^*P* < 0.05.

## Data Availability

The data used during the current study are available at the Secretariat, Higher Degrees, Research and Ethics Committee, School of Public Health, Makerere University and can be requested through the corresponding author.
